# Time Trends in Causes of Death in People With HIV: Insights From the Swiss HIV Cohort Study

**DOI:** 10.1093/cid/ciae014

**Published:** 2024-01-12

**Authors:** M S R Weber, J J Duran Ramirez, M Hentzien, M Cavassini, E Bernasconi, E Hofmann, H Furrer, H Kovari, M Stöckle, P Schmid, D Haerry, D L Braun, H F Günthard, K Kusejko, the Swiss HIV Cohort Study, I Abela, K Aebi-Popp, A Anagnostopoulos, M Battegay, E Bernasconi, D L Braun, H C Bucher, A Calmy, M Cavassini, A Ciuffi, G Dollenmaier, M Egger, L Elzi, J Fehr, J Fellay, H Furrer, C A Fux, H F Günthard, A Hachfeld, D Haerry, B Hasse, H H Hirsch, M Hoffmann, I Hösli, M Huber, D Jackson-Perry, C R Kahlert, O Keiser, T Klimkait, R D Kouyos, K Kusejko, N Labhardt, K Leuzinger, B Martinez de Tejada, C Marzolini, K J Metzner, N Müller, J Nemeth, D Nicca, J Notter, P Paioni, G Pantaleo, M Perreau, A Rauch, L Salazar-Vizcaya, P Schmid, R Speck, M Stöckle, P Tarr, A Trkola, G Wandeler, M Weisser, S Yerly

**Affiliations:** Department of Infectious Diseases and Hospital Epidemiology, University Hospital Zurich, Zurich, Switzerland; Institute of Medical Virology, University of Zurich, Zurich, Switzerland; Department of Infectious Diseases and Hospital Epidemiology, University Hospital Zurich, Zurich, Switzerland; Institute of Medical Virology, University of Zurich, Zurich, Switzerland; HIV/AIDS Unit, Department of Infectious Diseases, University Hospital Geneva, Geneva, Switzerland; Department of Medicine, University of Geneva, Geneva, Switzerland; UR3797, Reims Champagne-Ardenne University, Reims, France; Department of Infectious Diseases, Lausanne University Hospital, University of Lausanne, Lausanne, Switzerland; Department of Infectious Diseases, Regional Hospital Lugano EOC, University of Geneva and University of Southern Switzerland, Lugano, Switzerland; Department of Infectious Diseases, Inselspital, Bern University Hospital, University of Bern, Bern, Switzerland; Department of Infectious Diseases, Inselspital, Bern University Hospital, University of Bern, Bern, Switzerland; Center for Infectious Diseases, Klinik im Park, Zürich, Switzerland; Division of Infectious Diseases and Hospital Epidemiology, University Hospital Basel, University of Basel, Basel, Switzerland; Division of Infectious Diseases and Hospital Epidemiology, Cantonal Hospital St Gallen, St Gallen, Switzerland; Positive Council Switzerland, Zürich, Switzerland; Department of Infectious Diseases and Hospital Epidemiology, University Hospital Zurich, Zurich, Switzerland; Institute of Medical Virology, University of Zurich, Zurich, Switzerland; Department of Infectious Diseases and Hospital Epidemiology, University Hospital Zurich, Zurich, Switzerland; Institute of Medical Virology, University of Zurich, Zurich, Switzerland; Department of Infectious Diseases and Hospital Epidemiology, University Hospital Zurich, Zurich, Switzerland; Institute of Medical Virology, University of Zurich, Zurich, Switzerland

**Keywords:** HIV/AIDS, cause of death, cancer, hepatitis, cardiovascular risk

## Abstract

**Background:**

Advancements in access to antiretroviral therapy (ART) and human immunodeficiency virus (HIV) care have led to a decline in AIDS-related deaths among people with HIV (PWH) in Switzerland. However, data on the ongoing changes in causes of death among PWH over the past 15 years are scarce.

**Methods:**

We investigated all reported deaths in the Swiss HIV Cohort Study between 2005 and 2022. Causes of death were categorized using the Coding Causes of Death in HIV protocol. The statistical analysis included demographic stratification to identify time trends and logistic regression models to determine associated factors for the underlying cause of death.

**Results:**

In total, 1630 deaths were reported, with 23.7% of individuals assigned female sex at birth. These deaths included 147 (9.0%) HIV/AIDS-related deaths, 373 (22.9%) due to non-AIDS, non-hepatic cancers, 166 (10.2%) liver-related deaths, and 158 (9.7%) cardiovascular-related deaths. The median age at death (interquartile range) increased from 45.0 (40.0–53.0) years in 2005–2007 to 61.0 (56.0–69.5) years in 2020–2022. HIV/AIDS- and liver-related deaths decreased, whereas deaths from non-AIDS, non-hepatic cancers increased and cardiovascular-related deaths remained relatively stable.

**Conclusions:**

The proportionally decreasing HIV/AIDS and liver-related deaths showcase the effectiveness of ART, comprehensive HIV patient care, and interventions targeting hepatitis C virus coinfection. Future research should focus on managing cancer and cardiovascular-related conditions as the new leading causes of death among PWH. Comprehensive healthcare strategies focusing on non–AIDS-related comorbid conditions, cancer management, and sustaining liver and cardiovascular health are needed to bridge the ongoing health disparities between PWH and the general population.

## Teaser

In the Swiss HIV Cohort Study (2005–2022), human immunodeficiency virus/AIDS–related and liver-related deaths decreased proportionally, whereas cardiovascular-related deaths remained stable and non–AIDS-related cancers increased, underscoring the need for holistic healthcare strategies addressing comorbid all conditions.

The advent of combination antiretroviral therapy (ART) in 1996 transformed the cause of death landscape, substantially reducing AIDS-related mortality rates in people with human immunodeficiency virus (HIV; PWH), thus extending their life expectancy [1–4]. With successful virus suppression through ART, the cause of death profile of PWH shifted, with a rise in non–AIDS-defining cancer deaths, liver-related conditions, and cardiovascular diseases [1, 3–5]. However, despite these advancements, health disparities in PWH persist for some subgroups compared to the general population, even in an advanced healthcare systems like Switzerland's [2, 6]. While HIV-related factors like late-stage diagnosis, delayed ART initiation, ART-related side effects, low-level viral replication, and inflammation play a role, non–HIV-related factors, such as sociodemographic and behavioral differences (eg, higher rates of illicit drug use, smoking, and sexual behavior), also contribute significantly [7–9]. As a result, PWH experience higher rates of coinfections, including hepatitis C virus (HCV) and other oncogenic viruses, leading to higher rates of cardiovascular events and non–AIDS-defining cancers, such as lung cancer [1, 7–14].

In 1988, Switzerland faced the highest AIDS incidence rate in Europe and a significant prevalence of HCV coinfections, primarily linked to outbreaks among people who inject drugs (PWID) [15]. However, Switzerland made substantial progress evidenced by decreasing mortality rates and increasing life expectancy for PWH in the Swiss HIV Cohort Study (SHCS), and it is expected to achieve the UNAIDS 95-95-95 targets by 2030 [1, 6, 16]. Factors contributing to this progress include progressive drug policies, the establishment of the SHCS, a representative longitudinal study enrolling PWH in Switzerland, and the national SwissPrEPared program, aiming to improve medical care for people at increased risk of HIV, in combination with the presence of a robust public healthcare system [15, 17, 18]. These collective efforts facilitated comprehensive patient care while mitigating HIV and HCV acquisition among PWID and men who have sex with men (MSM), who emerged as the new primary demographic of HIV and HCV acquisition [14, 19–21]. Switzerland's unique HIV history thus provides an ideal setting to investigate the intricate dynamics of the HIV epidemic in the modern ART era.

In the face of this ever-evolving landscape, leading to an increasingly aging population of PWH, the necessity for up-to-date insights into causes of death among PWH remains crucial [4]. However, publications focusing on the ongoing changes in causes of death in PWH over the past 15 years are scarce [1, 4]. Therefore, we investigated longitudinal patterns in causes of death and associated factors among PWH enrolled in the SHCS between 2005 and 2022 to shed light on the evolving Swiss HIV epidemic and provide a foundation for future research in a global context.

## METHODS

### Swiss HIV Cohort Study

The SHCS, a national, longitudinal, multicenter cohort study has enrolled adult PWH since 1988 to monitor the HIV epidemic in Switzerland [17]. The study cumulatively includes 21 782 participants (database download 15 August 2023), of whom 6233 have died, 9412 are still active, and 6137 discontinued the study for other reasons. Detailed information on demographics, psychosocial factors, clinical data, laboratory results, and treatment is collected biannually. The SHCS documents HIV-associated diseases and causes of death since 1988 and non–AIDS-related cancers since 1999. Information on underlying causes of death have been collected since 2005, following the Coding Causes of Death in HIV (CoDe) protocol [22].

### Study Design

Our analysis included all reported deaths in the SHCS since the adoption of the CoDe protocol, from 1 January 2005 to 31 December 2022, including people who had previously dropped out of the study and deaths reported from alternative sources (eg, relatives, noncohort physicians, and hospital records). All deaths were included, regardless of how detailed the reported circumstances, as long as the year of death was known. Cases lacking sufficient information on the cause of death were labeled as unknown.

### Assignment of Causes of Death

For each reported death, a trained physician formed a narrative of the events leading to death using cause of death information provided by the treating clinician and incorporating the SHCS’s time-updated granular clinical, laboratory, demographic, and behavioral information. In cases where information was missing, queries were made to the treating physicians. In cases of ambiguity, a panel of experts, including a senior infectious disease physician, convened to reach consensus. The corresponding *International Statistical Classification of Diseases, Tenth Revision* (*ICD-10*) code was assigned to the underlying cause of death if a clear narrative could be formed. Based on the CoDe protocol, we translated the *ICD-10* codes into CoDe codes to group individual causes of death into categories (Table 1) [22].

**Table 1.** Cause of Death Translation Table of Broad Categories, Coding Causes of Death in HIV, and *International Statistical Classiﬁcation of Diseases* Codes^a^

Category	Included Conditions	CoDe Categories^b^	*ICD-10* Codes
HIV/AIDS	HIV/AIDS infections and cancers	01	A021, A072–073, A15–A19, A31, A812, B027, B20–B24, B25, B371, B383–B389, B393–B399, B451–B459, B582, C46, C53, C82, C83, C85
Non–AIDS infection	Infections other than AIDS-defining, opportunistic infections	02	A00–A020, A022–A071, A078–A09, A20–A309, A32–A812, A818–A99, B0–B09, B26–B370, B372–B382, B39–B392, B40–B450, B46–B581, B583–B941, B948–B99, G00–G02, I33.0, J01–J22, J85, M72.6, N39.0
NANH cancer	All cancers except AIDS defining or hepatic	04	C00–C45, C47–C52, C54–C81, C84, C88–D09
Liver	Chronic viral hepatitis, liver failure, and hepatic cancers	03, 14, 04.20	B15–B19, B942, K70–K77, C22.0
Cardiovascular/heart	Acute MI, stroke, and other diseases of the circulatory system	08, 09, 24, 11, 12	All other I
Respiratory	COPD and other respiratory diseases	13, 25	All other J
Substance use	Active substance use including acute intoxication	19	F10–F19, Y12
Violent death	Suicides, accidents, or other violent deaths	16, 17	V–X
CNS	CNS disease including Parkinson's and Alzheimer disease	23	G03–G99
Renal/urogenital	Renal failure, urogenital diseases	15, 28	All other N
GI tract	Pancreatitis, GI hemorrhage, digestive system diseases	06, 10, 26	All other K
Unknown/unclassifiable	Unclassifiable causes or unknown	91, 92	R09.2, R96–R99, unknown
Other	Other causes	05, 07, 20, 21, 22, 90	E, all other F, all other D

Abbreviations: CNS, central nervous system; CoDe, Coding Causes of Death in HIV; COPD, chronic obstructive pulmonary disease; GI, gastrointestinal; HIV, human immunodeficiency virus; *ICD-10, International Statistical Classification of Diseases, Tenth Revision*; MI, myocardial infarction; NANH, non-AIDS, nonhepatic.

^a^Individual causes of death were converted into broader categories for time trends and associated factor analysis, adapted from Weber et al [1].

^b^CoDe protocol (version 2.3; Copenhagen HIV Program [CHIP]) [22].

### Statistical Analysis

We described total numbers and fractions of the cause of death categories, stratified by calendar year period. We used 4 logistic regression models to identify and quantify associated factors with (1) AIDS-related, (2) liver-related, (3) non-AIDS, nonhepatic (NANH) cancer, and (4) cardiovascular-related deaths, respectively, versus deaths from other causes. Covariables included in the multivariable models were selected based on their significance in univariable analysis (*P* < .05) and clinical relevance (Table 2). Data analysis was conducted using R-Studio software, version 4.3.0 (21 April 2023).

**Table 2.** Definitions of Variables^a^

Variables	Definition
Age at death	Categories: <39, 40–49, 50–59, 60–70, and >70 y
Sex and acquisition mode	Categories: heterosexual men, heterosexual women, MSM, men who inject drugs, women who inject drugs, other men, and other women
Race/ethnicity	Categories: white, black, Hispano-American, Asian, other, and unknown
Higher educational or university degree	Defined as completed higher educational or university degree above the mandatory 9-y school period, apprenticeship, or high school; categories: yes or no
Nadir CD4 cell count	Lowest CD4 cell count ever measured >6 mo before death; categories: <50/μL, 50–99/μL, 100–199/μL, 200–349/μL, and ≥350/μL
Time since HIV diagnosis	Defined as the time between HIV diagnosis and death in years, as a proxy for changing treatment regimens, guidelines over time, and the impact of long-term ART exposure or exposure to the HIV virus itself
HIV viral load	Quantified by calculating the AUC normalized for time, as a proxy for the impact of viral replication irrespective of time since HIV diagnosis
Prior clinical AIDS	Defined as any prior diagnosis of a CDC HIV category B/C event; categories: yes or no
ART at death	Defined as reported being on ART at time of death; categories: ART naive, on ART, and ART interrupted >1 mo before death
Smoking status	Defined as having reported ever smoking, quantified in pack-years
Diabetes mellitus	Diagnosis of diabetes mellitus of any type or elevated HbA_1c_ measurement of >6.5% at any follow-up or taking diabetic medication; categories: yes or no
Hypertension	Defined as 2 consecutive elevated blood pressure measures of >140 mm Hg systolic or >90 mmHg diastolic or/and taking hypertensive medication; categories: yes or no
Hypercholesterolemia	Defined as 2 consecutive elevated LDL-C measurements of >3 mmol/L or/and taking lipid-lowering medication; categories: yes or no
Prior cardiovascular event	Defined as reporting ≥1 of the following events: MI, coronary angioplasty/stenting, coronary artery bypass, cerebral infarction, carotid endarterectomy, or procedures on other arteries; categories: yes or no
BMI	BMI in last follow-up >6 mo before death; categories: underweight (<18.5), normal weight (18.5–24.9), overweight (25–29.9), and obese (≥30)^b^
Excessive alcohol consumption	Defined as any report of daily alcohol consumption >40 g/d or an AUDIT-C score ≥3 in female or ≥4 in male participants; categories: yes or no
HCV coinfection	Defined by positive HCV RNA detection at any follow-up; categories: yes or no
HBV coinfection	Defined by positive HBV DNA or HBsAg or HBeAg detected at any follow-up; categories: yes or no
CMV coinfection	Defined by positive CMV IgG detected at any follow-up; categories: yes or no
Depression	Defined as self-reported symptoms of depression; categories: yes or no
3-y Periods	Intervals chosen to maintain an adequate sample size, while roughly aligning with key changes in PWH treatment guidelines in Switzerland; categories: 2005–2007 (before Swiss Statement and the guideline to start ART independent of the CD4 cell count [‘treat-all’]), 2008–2010 (rollout of treat-all guidelines following the Swiss Statement), 2011–2013 (well-established treat-all guidelines), 2014–2016 (introduction of highly effective DAAs in HCV management in Switzerland), 2017–2019 (nationwide universal DAA access regardless of liver failure status), 2020–2022 (COVID-19 pandemic)

Abbreviations: ART, antiretroviral therapy; AUC, area under the curve; AUDIT-C, Alcohol Use Disorder Identiﬁcation Test Consumption; BMI, body mass index; CDC, Centers for Disease Control and Prevention; CMV, cytomegalovirus; COVID-19, coronavirus disease 2019; DAA, direct-acting agent; HbA_1c_, hemoglobin A_1c_; HBeAg, hepatitis B e antigen; HBsAg, hepatitis B surface antigen; HBV, hepatitis B virus; HCV, hepatitis C virus; HIV, human immunodeficiency virus; IgG, immunoglobulin G; LDL-C, low-density lipoprotein cholesterol; MI, myocardial infarction; MSM, men who have sex with men; PWH, people with HIV.

^a^Definition and categories of all clinical, behavioral, and sociodemographic variables used in each univariable logistic regression analysis and for patient characterization.

^b^BMI calculated as weight in kilograms divided by height in meters squared.

## RESULTS

### Patient Characteristics

A total of 1630 deaths occurred in the SHCS between 1 January 2005 and 31 December 2022, 386 (23.7%) in persons assigned female sex at birth. The median age at death was 54.0 (interquartile range [IQR], 46.0–63.0) years, increasing from 45.0 (40.0–53.0) years in 2005–2007 to 61.0 (56.0–69.5) years in 2020–2022 and was lowest for deaths due to overdose of narcotics, at 44.0 (40.0–48.0) years, and highest for deaths related to the central nervous system, at 75.0 (59.0–78.5) years (Supplementary Table 1). The median follow-up time (IQR) within the SHCS was 13.4 (7.5–19.7) years, while the median time between HIV diagnosis and death was 17.0 (11.0–24.0) years. The proportion of deceased PWID decreased from 46.4% (140 of 302) in 2005–2007 to 22.5% (60 of 267) in 2020–2022, whereas the proportion of deceased MSM increased from 22.8% (69 of 302) in 2005–2007 to 39.3% (105 of 267) in 2020–2022 (Table 3).

**Table 3.** Patient Characteristics by 3-Year Periods^a^

Characteristic	Patients, No. (%)						
	Overall (N = 1630)	2005–2007 (n = 302)	2008–2010 (n = 285)	2011–2013 (n = 263)	2014–2016 (n = 269)	2017–2019 (n = 244)	2020–2022 (n = 267)
Age at death, median (IQR), y	54.00 (46.00– 63.00)	45.00 (40.00–53.00)	49.00 (44.00–59.00)	52.00 (46.50–60.00)	55.00 (49.00–66.00)	60.00 (54.00–67.00)	61.00 (56.00–69.50)
Follow-up time, median (IQR), y	13.39 (7.53–19.74)	8.88 (4.62–12.23)	11.61 (6.45–15.58)	13.47 (7.32–18.57)	16.28 (9.71–20.15)	17.99 (9.66–22.75)	21.15 (12.43–27.07)
Assigned female at birth	386 (23.7)	75 (24.8)	75 (26.3)	66 (25.1)	61 (22.7)	51 (20.9)	58 (21.7)
HIV acquisition mode							
MSM	506 (31.0)	69 (22.8)	72 (25.3)	79 (30.0)	96 (35.7)	85 (34.8)	105 (39.3)
Heterosexual contact	488 (29.9)	81 (26.8)	83 (29.1)	78 (29.7)	70 (26.0)	81 (33.2)	95 (35.6)
PWID	571 (35.0)	140 (46.4)	116 (40.7)	97 (36.9)	92 (34.2)	66 (27.0)	60 (22.5)
Other	65 (4.0)	12 (4.0)	14 (4.9)	9 (3.4)	11 (4.1)	12 (4.9)	7 (2.6)
Race/ethnicity							
White	1499 (92.0)	274 (90.7)	259 (90.9)	249 (94.7)	252 (93.7)	216 (88.5)	249 (93.3)
Black	74 (4.5)	12 (4.0)	14 (4.9)	7 (2.7)	7 (2.6)	21 (8.6)	13 (4.9)
Hispano-American	15 (0.9)	3 (1.0)	4 (1.4)	1 (0.4)	3 (1.1)	1 (0.4)	3 (1.1)
Asian	25 (1.5)	7 (2.3)	5 (1.8)	6 (2.3)	2 (0.7)	4 (1.6)	1 (0.4)
Other/unknown	17 (1.0)	6 (2.0)	3 (1.1)	0 (0.0)	5 (1.9)	2 (0.8)	1 (0.4)
Higher education or university degree	314 (19.3)	41 (13.6)	56 (19.6)	47 (17.9)	52 (19.3)	63 (25.8)	55 (20.6)
Time since HIV diagnosis, median (IQR), y	17.00 (11.00–24.00)	12.00 (8.00–17.00)	15.00 (10.00–21.00)	17.00 (11.00–23.50)	19.00 (13.00–24.00)	21.00 (13.00–27.00)	24.00 (15.00–31.00)
CD4 cell count nadir, median (IQR), cells/μL	128.00 (48.00–230.00)	115.00 (43.50–235.00)	109.00 (39.00–216.00)	129.50 (49.00–217.75)	135.00 (58.00–239.00)	143.00 (61.50–236.75)	133.50 (47.25–236.25)
Prior clinical AIDS	673 (41.3)	126 (41.7)	121 (42.5)	109 (41.4)	102 (37.9)	97 (39.8)	118 (44.2)
ART duration, median, (IQR), y	13.00 (8.00–19.00)	9.00 (5.00–11.00)	12.00 (7.00–14.00)	13.50 (8.00–16.25)	17.00 (9.25–19.00)	18.00 (10.00–22.00)	22.00 (12.00–25.00)
On ART at time of death	985 (60.4)	139 (46.0)	133 (46.7)	140 (53.2)	151 (56.1)	193 (79.1)	229 (85.8)
Ever smoker	1265 (77.6)	238 (78.8)	223 (78.2)	214 (81.4)	217 (80.7)	176 (72.1)	197 (73.8)
Hypertension	920 (56.4)	110 (36.4)	133 (46.7)	152 (57.8)	173 (64.3)	164 (67.2)	188 (70.4)
BMI, median (IQR)^b^	22.39 (19.49–25.36)	21.46 (19.39–24.21)	21.62 (18.88–24.71)	22.16 (19.58–25.32)	22.60 (19.70–25.95)	23.05 (20.17–25.52)	23.19 (20.19–26.48)
Diabetes mellitus	195 (12.0)	25 (8.3)	29 (10.2)	37 (14.1)	33 (12.3)	34 (13.9)	37 (13.9)
Prior cardiovascular event	230 (14.1)	26 (8.6)	29 (10.2)	34 (12.9)	47 (17.5)	47 (19.3)	47 (17.6)
Hypercholesterolemia	967 (59.3)	121 (40.1)	141 (49.5)	149 (56.7)	169 (62.8)	174 (71.3)	213 (79.8)
Depression	574 (35.2)	Not available	67 (23.5)	112 (42.6)	131 (48.7)	123 (50.4)	140 (52.4)
HCV coinfection	524 (32.1)	111 (36.8)	98 (34.4)	90 (34.2)	91 (33.8)	71 (29.1)	63 (23.6)
HBV coinfection	137 (8.4)	22 (7.3)	30 (10.5)	18 (6.8)	21 (7.8)	23 (9.4)	23 (8.6)
CMV coinfection	1304 (80.0)	228 (75.5)	231 (81.1)	217 (82.5)	217 (80.7)	193 (79.1)	218 (81.6)

Abbreviations: ART, antiretroviral therapy; BMI, body mass index; CMV, cytomegalovirus; HBV, hepatitis B virus; HCV, hepatitis C virus; HIV, human immunodeficiency virus; IQR, interquartile range; MSM, men who have sex with men; PWID, persons who inject drugs.

^a^Patients’ basic, clinical, and laboratory characteristics overall and stratified by 3-year periods with the definition of all variables found in Table 2.

^b^BMI calculated as weight in kilograms divided by height in meters squared.

### Time Trends in Causes of Death

**Figure 1:**
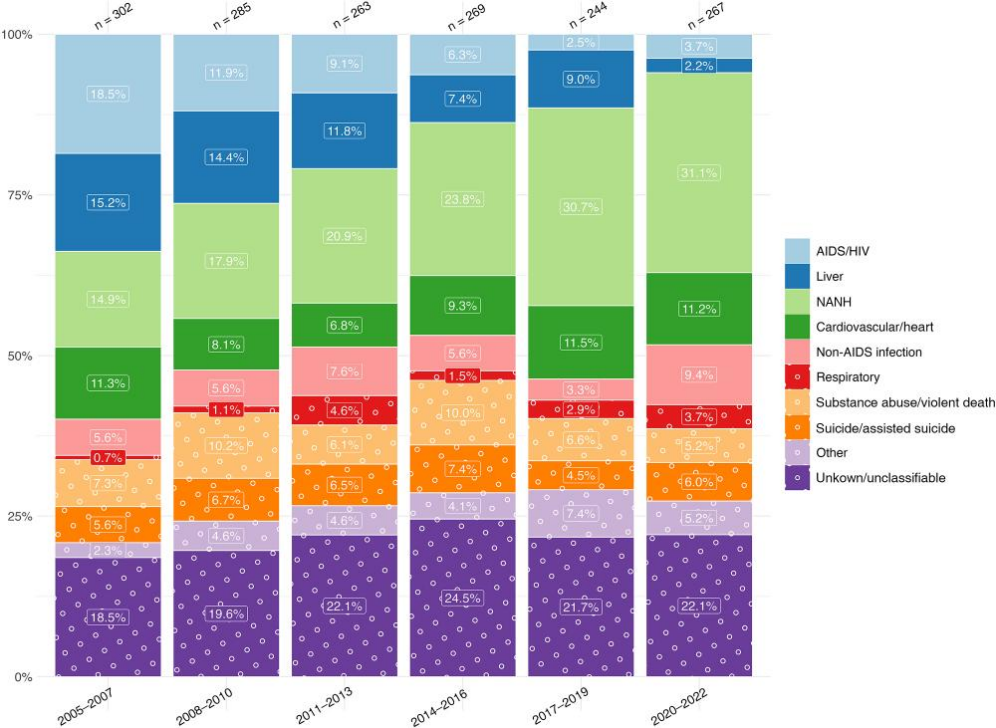
Time trends in causes of death from 2005 to 2022, stratified by 3-year periods. Single causes of death are categorized into broader categories as outlined in Table 1. The x-axis includes time periods from 2005 to 2022, grouped into 3-year intervals; y-axis, the percentage distribution for each cause-of-death category; number above bar, the total reported deaths for the corresponding 3-year period. Abbreviations: HIV, human immunodeficiency virus; NANH, non-AIDS, non-hepatic.

HIV/AIDS-related causes of death witnessed the most pronounced change in proportion, decreasing from 18.5% (56 of 302) in 2005–2007 to 3.7% (10 of 267) in 2020–2022, with liver-related causes decreasing from 15.2% (46 of 302) in 2005–2007 to 2.2% (6 of 267) in 2020–2022. In contrast, NANH cancers increased in proportion from 14.9% (45 of 302) in 2005–2007 to 31.1% (83 of 267) in 2020–2022, while cardiovascular-related diseases remained relatively stable, comprising 11.3% (34 of 302) of deaths in 2005–2007 and 11.2% (30 of 267) in 2020–2022 (Figure 1).

### HIV/AIDS-Related Causes of Death

**Figure 2.**
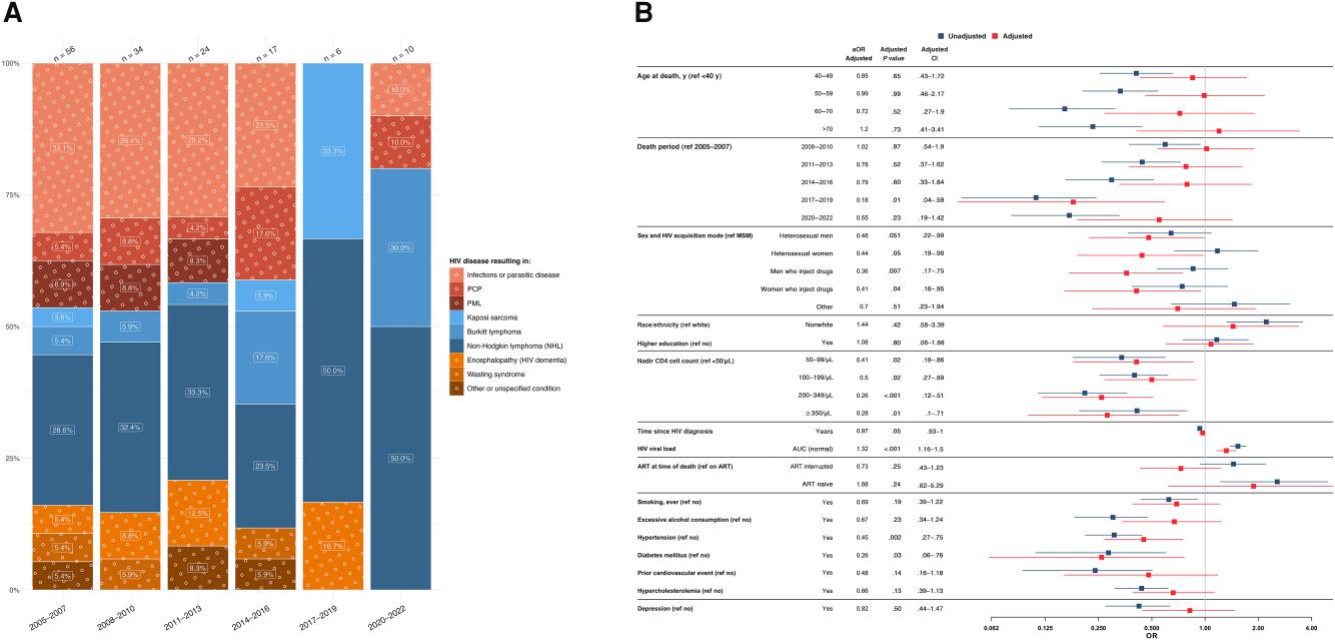
*A*, Time trends in human immunodeficiency virus (HIV)/AIDS–related causes of death from 2005 to 2022, stratified by 3-year periods. Single causes of death are categorized into broader categories, as outlined in Table 1, and further grouped into infectious causes (*red*), cancers (*blue*), and other conditions (*orange*). The x-axis includes time periods from 2005 to 2022, grouped into 3-year intervals; y-axis, the percentage distribution for each cause-of-death category; numbers above bars, the total reported deaths for the corresponding 3-year period. The number within each bar represents the percentage of each cause of death within its respective 3-year interval. *B*, Factors associated with HIV/AIDS-related causes of death. The y-axis includes all factors included in the multivariable/adjusted logistic regression analysis (*red*) based on their statistical significance in the univariable/unadjusted logistic regression analysis (*blue*) as well as clinical relevance; the x-axis, the odds for each factor, compared with its reference (ref) factor, of dying of an HIV/AIDS-related cause of death compared with any other cause of death. Exercise caution when interpreting this analysis, since certain factors may influence various causes of death, while others may specifically increase the odds of one particular cause of death. Definitions for all variables used in the univariable analysis are available in Table 2. Single death causes are categorized into broader causes, as outlined in Table 1. Abbreviations: aOR, adjusted odds ratio; ART, antiretroviral therapy; AUC, area under the curve; CI, confidence interval; MSM, men who have sex with men; NHL, non-Hodgkin lymphoma; OR, odds ratio; PCP, *Pneumocystis jirovecii* pneumonia; PML, progressive multifocal leukoencephalopathy.

A total of 147 (9.0%) deaths were attributed to HIV/AIDS-related causes; of those, 61 (41.5%) were attributed to AIDS-defining opportunistic infections, 64 (43.5%) to AIDS-defining cancer, and 22 (15.0%) to other AIDS-defining conditions. HIV/AIDS-related deaths attributable to infectious causes decreased from 46.4% in 2005–2007 to 20% in 2020–2022 (Figure 2*A*).

HIV acquisition through heterosexual contacts (adjusted odds ratio [aOR], 0.48 [95% confidence interval (CI), .22–.99] for male and 0.44 [.19–.98] for female participants) and intravenous drug use (0.36 [.17–.75] for male and 0.41 [.16–.95], for female participants) were associated with lower odds of HIV/AIDS-related death than in MSM. A nadir CD4 cell count ≥50/μL was associated with lower odds of HIV/AIDS-related deaths (aOR for CD4 cell count 200–349/μL, 0.26 [95% CI, .12–.51]), as was diabetes (0.26 [.06–.76]) and hypertension (0.45 [.27–.75]). A higher cumulative HIV viral load (aOR, 1.32 [95% CI, 1.16–1.5]) was associated with higher odds of such death, while no significant associations with other factors—including age at death, time since HIV diagnosis, ART status, and coinfections—were detected (Figure 2*B*).

Among the 10 individuals who died of HIV/AIDS-related causes between 2020–2022, the median age at death (IQR) was 55.0 (53.3–60.0) years, the median time since HIV diagnosis was 24.5 (8.8–30.8) years, with a median follow-up time in the SHCS of 17 (2.0–24.7) years. All 10 participants were white, of whom 8 reported receiving ART in the month before death. Eight of these deaths were from Burkitt lymphoma (5 of 10) or other types of non-Hodgkin lymphoma (NHL) (3 of 10). One death resulted from pneumocystis pneumonia, another from bacterial infection, while the CD4 cell count in the year of death was <100/μL (Supplementary Table 2).

### Liver-Related Causes of Death

A total of 166 deaths (10.2%) were due to liver-related conditions, the most frequent causes being HCV with cirrhosis (n = 77 [46.4%]), HCV with hepatocellular carcinoma (n = 42 [25.3%]), and HCV with liver failure (n = 14 [8.4%]). Hepatitis B virus (HBV) with cirrhosis accounted for 8 cases (4.8%), HBV with hepatocellular carcinoma for 7 cases (4.2%), HBV with liver failure for 2 cases (1.2%), while liver failure not due to chronic viral hepatitis accounted for 16 cases (9.6%).

**Figure 3.**
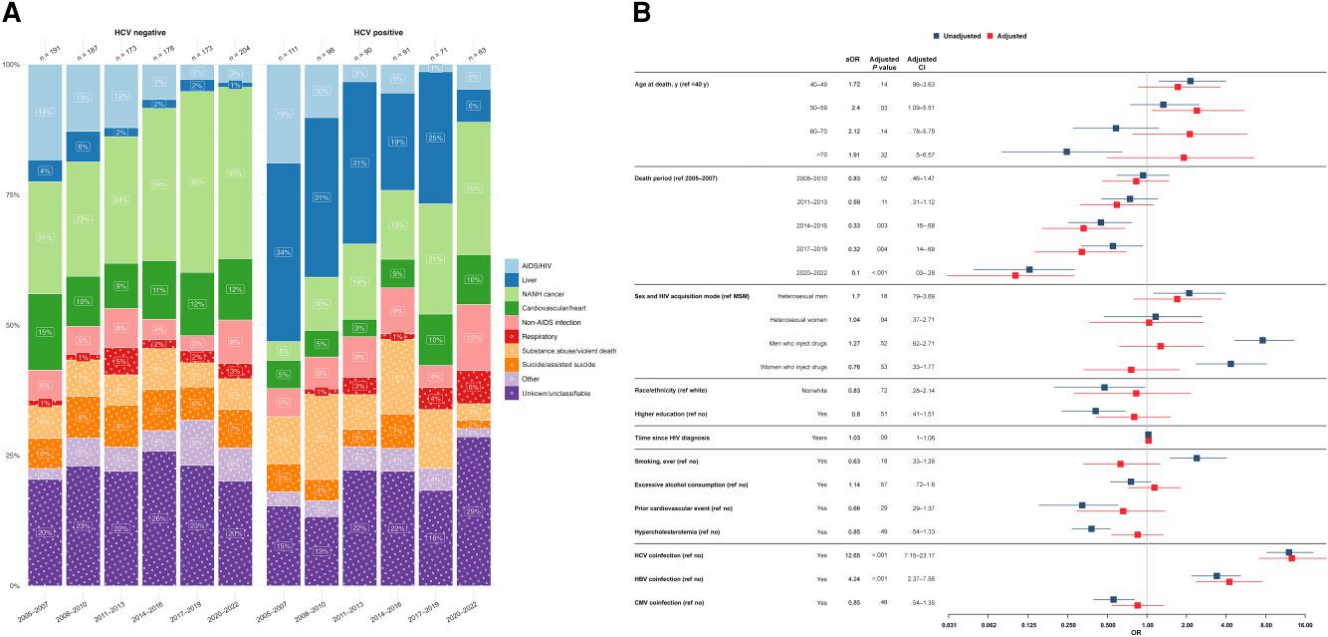
*A*, Time trends in causes of death grouped by hepatitis C virus (HCV) status from 2005 to 2022, stratified by 3-year periods. Single causes of death are categorized into broader categories as outlined in Table 1. *Left,* Time trends in causes of death among individuals without HCV coinfection. *Right,* Time trends in causes of death among individuals with HCV coinfection, defined as s positive HCV RNA result at any follow-up. The x-axis includes 3-year intervals from 2005 to 2022; y-axis; percentage distribution of each cause of death category; numbers above bars, the total reported deaths for the corresponding 3-year period. The number within each bar indicates the percentage of each cause of death within its respective 3-year period. *B*, Factors associated with liver-related causes of death. The y-axis includes all factors included in the multivariable/adjusted logistic regression analysis (*red*), based on their statistical significance in the univariable/unadjusted logistic regression analysis (*blue*) as well as clinical relevance; the x-axis, the odds for each factor, compared with its reference (ref) factor, of dying of a liver-related cause of death compared with any other cause. Exercise caution when interpreting this analysis, since certain factors may influence various causes of death, while others may specifically increase the odds of one particular cause of death. Definitions for all variables used in the univariable analysis are available in Table 2. Single death causes are categorized into broader causes. as outlined in Table 1. Abbreviations: aOR, adjusted odds ratio; ART, antiretroviral therapy; CI, confidence interval; CMV, cytomegalovirus; HBV, hepatitis B virus; HIV, human immunodeficiency virus; MSM, men who have sex with men; NANH, non-AIDS, nonhepatic; OR, odds ratio.

Most liver-related deaths occurred in people with HCV coinfection (n = 135 [80.1%]). A comparison of causes of death between people without HCV coinfection (n = 1106 [67.9%]) and those with HCV coinfection (n = 524 [32.1%]) revealed that in 2005–2007, HIV/AIDS-related causes were most prevalent in people without HCV coinfection (35 of 191 [18.3%]), while in people with HCV coinfection, liver-related conditions emerged as the predominant causes of death (38 of 111 [34.2%]). Over time, both groups showed significant proportional reductions in liver-related and HIV/AIDS-related deaths, with NANH cancers emerging as the leading cause of death (Figure 3*A*; Supplementary Figure 1).

Participants with HCV coinfection had significantly elevated odds of liver-related death (aOR, 12.65 [95% CI, 7.15–23.17]), as did those with HBV coinfection (4.24 [2.37–7.56]). Moreover, individuals who died before 2013 showed notably higher odds of liver-related death than those who died in subsequent years (aOR for deaths in 2020–2022, 0.10 [95% CI, .03–.28]). In addition, participants aged 50–59 years had higher odds of liver-related death than those in the <40-year age group (aOR, 2.40 [95% CI, 1.09–5.51]). No significant associations were found between liver-related death, sex and acquisition mode, cardiovascular risk factors, alcohol consumption, and ART status (Figure 3*B*).

### NANH Cancer–Related Causes of Death

**Figure 4.**
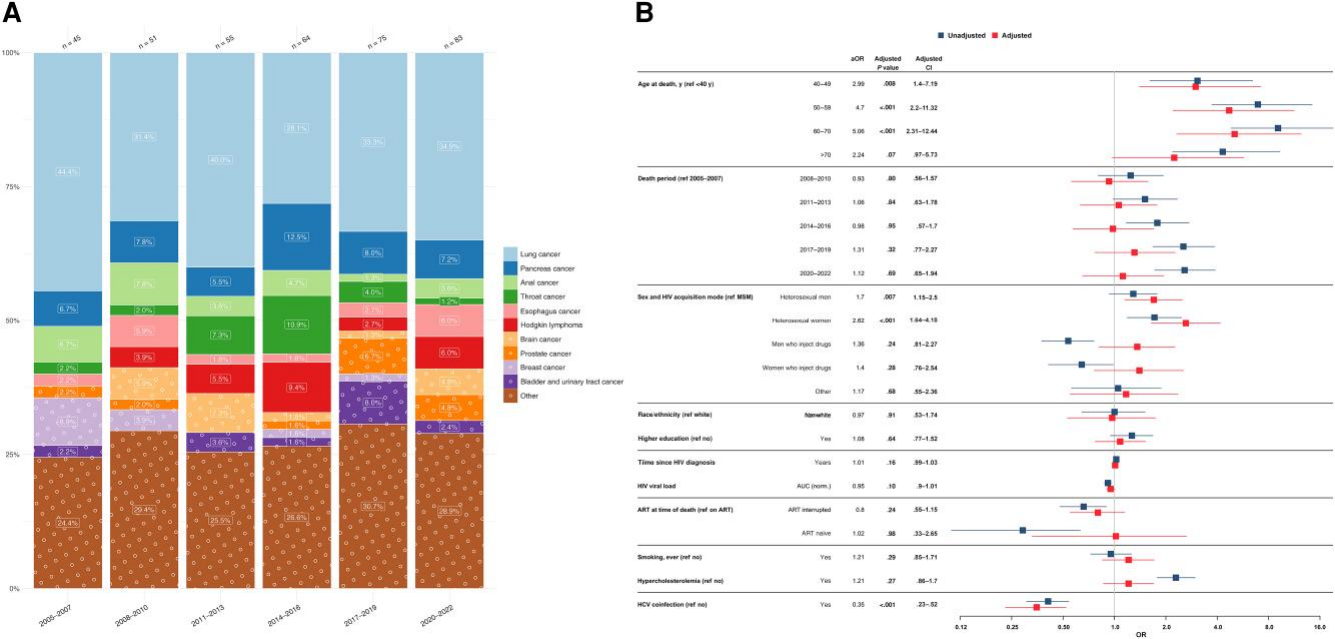
*A*, Time trends in non-AIDS, nonhepatic (NANH) cancer–related causes of death from 2005 to 2022, stratified by 3-year periods. Single causes of death are categorized into broader categories, as outlined in Table 1. The x-axis includes 3-year intervals from 2005–2022; y-axis, the percentage distribution for each cause of death; numbers above bars, total reported deaths for the corresponding 3-year period. The number within each bar indicates the percentage for each cause of death within its 3-year interval. *B*, Factors associated with NANH cancer–related causes of death. The y-axis includes all factors included in the multivariable/adjusted logistic regression analysis (*red*), based on their statistical significance and the univariable/unadjusted logistic regression analysis (*blue*) and clinical relevance. The x-axis shows the odds for each factor, compared with its reference factor (ref), of dying of an NANH cancer–related cause of death compared with any other causes of death. Exercise caution when interpreting this analysis, since certain factors may influence various causes of death, while others may specifically increase the odds of one particular death cause. Definitions for all variables used in the univariable analysis are available in Table 2. Single death causes are categorized into broader causes, as outlined in Table 1. Abbreviations: aOR, adjusted odds ratio: ART, antiretrovial therapy; AUC, area under the curve; CI, confidence interval; HCV, hepatitis C virus; HIV, human immunodeficiency virus; OR, odds ratio.

A total of 373 (22.9%) deaths were attributed to NANH cancers, varying from 11 (14.3%) to 32 (29.3%) cases per year, with overall increasing proportions. Lung cancers consistently emerged as the most frequent group, accounting for 130 cases (34.9%), followed by pancreatic cancers, with 30 cases (8.0%) (Figure 4*A*).

Individuals with NANH cancer–related deaths had higher odds of having acquired HIV through heterosexual contact (aOR, 1.70 [95% CI, 1.15–2.50] for male and 2.62 [1.64–4.18] for female participants) than MSM. Older age was associated with higher odds of NANH cancer–related death, with increasing odds for subsequent age groups compared with those aged <40 years (aOR for the 60–70-year age group, 5.06 [95% CI, 2.31–12.44]). Conversely, participants with NANH cancer–related deaths had reduced odds of having HCV coinfections (aOR, 0.35 [95% CI, .23–.52]) compared with those dying of other causes. No significant associations were detected between NANH cancer–related deaths, calendar period, cardiovascular risk factors, HIV viral load, or nadir CD4 cell count (Figure 4*B*).

We selected the most prevalent group, those with lung cancer–related deaths, for in-depth associated factor analysis. HIV acquisition through heterosexual contacts was associated with higher odds of lung cancer–related death than in MSM (aOR, 1.77 [95% CI, 1.01–3.11] in male and 2.7 [1.37–5.25] in female participants). Individuals aged 50–70 years displayed increased odds of lung cancer–related death compared with those aged <40 years (aOR for participants aged 50–59 years, 3.45 [95% CI, 1.21–12.6]), while a smoking history of >20 pack-years demonstrated a clear association with increased odds of lung cancer–related death (aOR for history of ≥60 pack-years, 4.46 [95% CI, 1.97–10.22]). Higher HIV viral load (aOR, 0.9 [95% CI, .83–.98]) and HCV coinfection (0.37 [.20,–67]) were associated with lower odds of lung cancer–related death, compared with all other death causes, while no significant associations were found between lung cancer–related deaths and various factors, including calendar period or ART at death (Supplementary Figure 2).

### Cardiovascular/Heart-Related Causes of Death

**Figure 5.**
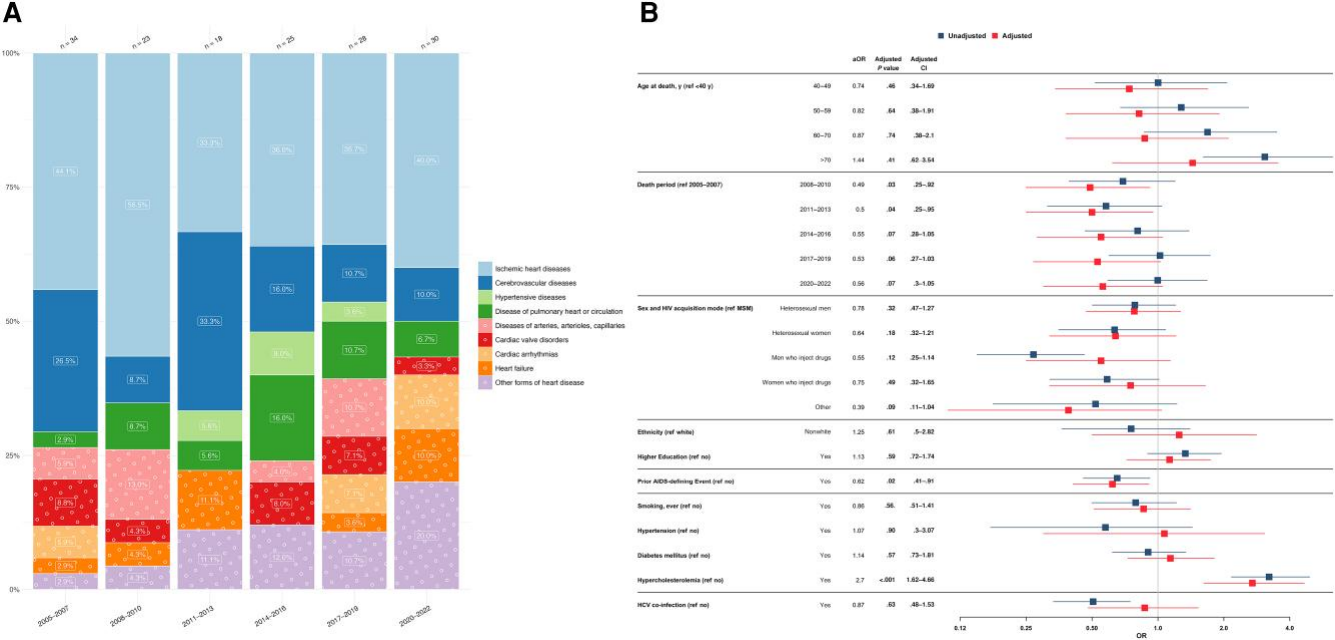
*A*, Cardiovascular/heart-related causes of death from 2005 to 2022, stratified by 3-year periods. Single causes of death are grouped into broader categories as outlined in Table 1. The x-axis includes the 3-year intervals spanning from 2005 to 2022; y-axis, the percentage distribution for each cause of death; numbers above bars, the total reported deaths for the corresponding 3-year period. The number within each bar represents the percentage of each cause of death within its respective 3-year interval. *B*, Factors associated with cardiovascular/heart-related causes of death. The y-axis includes all factors in the multivariable/adjusted logistic regression analysis (*red*), based on their statistical significance in the univariable/unadjusted logistic regression analysis (*blue*) as well as clinical relevance; x-axis, the odds for each factor, compared with its reference factor (ref), of dying of a cardiovascular/heart-related cause of death compared with any other causes of death. Exercise caution when interpreting this analysis, since certain factors may influence various causes of death, while others may specifically increase the odds of one particular cause of death. Definitions for all variables used in the univariable analysis are available in Table 2. Single death causes are categorized into broader causes, as outlined in Table 1. Abbreviations: aOR, odds ratio; CI, confidence interval HCV, hepatitis C virus; HIV, human immunodeficiency virus; MSM, men who have sex with men; OR, odds ratio.

A total of 158 deaths (9.7%) were attributed to cardiovascular/heart-related conditions, varying from 5 (6.2%) to 17 (16.0%) cases per year, with an overall stable trend. Ischemic heart disease emerged frequent, with 65 cases (41.1%), followed by cerebrovascular diseases, which accounted for 27 deaths (17.1%) (Figure 5*A*).

Participants with hypercholesterolemia had significantly higher odds of cardiovascular/heart-related death (aOR, 2.7 [95% CI, 1.62–4.66]), while no significance was found for hypertension or diabetes. In addition, individuals who died before 2014 exhibited lower odds of cardiovascular-related death than those who died in 2005–2007 (aOR for deaths in 2008–2010, 0.49 [95% CI, .25–.92]). No significant associations were found between cardiovascular-related deaths, age at death, HIV acquisition mode, HIV viral load, or other cardiovascular risk factors compared with all other death causes (Figure 5*B*).

## DISCUSSION

HIV patient care has improved over the past 2 decades, reflected by increasing median age at death (from 44 to 62 years) and proportional decline in HIV/AIDS-related and liver-related deaths. In contrast, NANH cancer–related deaths increased in proportion, while cardiovascular-related deaths remained relatively stable. The shift to non–AIDS-related causes is in line with findings from other cohort studies, although the proportions of HIV/AIDS-related deaths are lower than in other high-income countries, underscoring the success of HIV patient care in Switzerland [4, 23, 24]. Furthermore, the decline in HIV/AIDS-related deaths after 2009, following previous work from Weber and colleagues [1] assessing death causes in the SHCS, highlights the benefit of early and universal ART initiation [25–27]. This decline is especially pronounced for HIV/AIDS-related deaths attributed to infectious causes, reflecting improvements in preventing opportunistic infections in PWH. Among the 10 individuals who died of HIV/AIDS-related causes in 2020–2022, 8 succumbed to Burkitt lymphoma or other NHL. Because NHL also occurs in HIV-negative individuals, albeit less frequently, the remaining HIV/AIDS-related lymphoma deaths in Switzerland could roughly correspond to the prevalence of NHL in the general Swiss population, although our study lacks the power to definitively establish this [28–30].

Overall, liver-related deaths declined in proportion, with the strongest decline seen in PWID and MSM, coinciding with higher HCV coinfection rates in these groups. Of note, an increase in HCV was observed among MSM after 2008, coinciding with an epidemic related to chemsex practices and increasing proportions of MSM engaging in condomless sex [21]. This decline is attributed to the introduction of effective direct-acting antiviral agents in the 2010s, linked with holistic harm-reduction programs, epidemic monitoring, and targeted interventions, such as the nationwide HCV microelimination program in 2016 targeting MSM in the SHCS [1, 15, 19, 20]. However, despite these interventions, HCV and HBV coinfection remained the main associated factors for liver-related deaths [1, 7]. Our analysis showed no correlation between liver-related deaths and excessive alcohol consumption, likely owing to the less pronounced impact of alcohol on liver-related deaths compared to more prominent influences of HBV and HCV coinfections and the potential toxicity of some ART medications [31]. Furthermore, we could not confirm the association between hypertension or diabetes and liver-related deaths reported by the Data Collection on Adverse Events of Anti-HIV Drugs (D:A:D) study, indicating the effective management of these cardiovascular risk factors within the SHCS [7, 32].

The substantial progress in reducing HIV/AIDS-related and liver-related deaths led to a gradual alignment of death causes with the general population, namely cardiovascular conditions and NANH cancer [33]. NANH cancer–related deaths doubled in proportion from 2005 to 2022, mainly attributable to improvements regarding HIV/AIDS- and liver-related deaths. This is in line with trends reported in other studies and also reflected by the aging population of PWH [1, 4, 24]. Interestingly, participants who reported heterosexual HIV acquisition mode had higher odds of dying of NANH cancers than MSM. Given the diverse genesis of cancer entities, we focused on the most prevalent group, lung cancers. Interestingly, female participants with heterosexual HIV acquisition mode had the highest odds of dying of lung cancer. However, the underlying association remains unclear. Furthermore, we identified a substantial dose-response between smoking exceeding 20 pack-years and the odds of dying of lung cancer. This aligns with previously published findings, thus reinforcing the robustness of our model. Remarkably, while the D:A:D study found a significant association between smoking and non-AIDS cancers in general, our study found an association only for lung cancers in PWH but not all non-AIDS cancers, highlighting the need for investigating different cancer types separately because they are etiologically incomparable [7].

We found stable proportions of cardiovascular-related deaths despite an aging study population [11, 12, 34, 35]. This is likely due to the extensive cardiovascular risk management used in the SHCS, effectively addressing hypertension and diabetes [35–37]. However, it is essential to continue focusing on managing preventable risk factors common to all cardiovascular conditions. This includes treating hypercholesterolemia and minimizing the potential toxicity of long-term ART exposure to protect cardiovascular health. Therefore, promoting healthy lifestyles, such as physical activity, along with lipid-lowering medication, remains crucial for reducing the burden of hypercholesterolemia on cardiovascular health in PWH [32, 38].

Note that our study is centered on causes of death and does not address mortality rates. While interpreting the associated factor analysis describing the odds of one death outcome compared with all others, certain factors, such as HCV coinfection, distinctly elevate the odds of a particular outcome (ie, liver-related deaths), while other factors like smoking affect the odds across multiple outcomes (ie, cardiovascular and lung cancer–related deaths). Determining the underlying cause of death is inherently challenging, but our study benefits from the SHCS's detailed longitudinal reporting process and adherence to the internationally recognized CoDe protocol [22]. However, for certain death causes, such as cardiovascular diseases, there may be underreporting, particularly when individuals died outside healthcare facilities [1, 39]. Nonetheless, there is no reason to assume this bias changed over time. Moreover, certain conditions requiring constant care, like Parkinson or Alzheimer disease, may lead to individuals leaving the SHCS for logistic reasons [1].

Despite concerns of potential disruptions from the coronavirus disease 2019 pandemic, annual patient attendance at follow-ups in the SHCS remained consistent, while our database download on 15 August 2023 ensured inclusion of potentially delayed reported data. Of note, the median age at death in the SHCS was significantly lower for men and women compared with the general Swiss population in 2018, making it challenging to compare death patterns owing to variations in demographics, behavior, and coinfections [33]. To draw such a comparison, fine-grained data on the general population would be necessary, which is beyond the scope of this study. Switzerland’s distinct approach to monitoring and managing its HIV epidemic, within a single robust healthcare system, provides the ideal setting to study the challenges faced by PWH in the modern ART era, since differences in healthcare access, risk factors, and demographics among countries contribute to varying disease burdens, distorting reported death causes of cohorts from different regions [40].

In summary, our study highlights 2 significant achievements in overcoming key challenges facing PWH in the modern ART era: the continued declining proportion of HIV/AIDS-related and liver-related deaths due to effective ART, comprehensive PWH patient care, and successful interventions targeting HCV coinfection. Future research should focus on NANH cancers and cardiovascular-related conditions as the new leading death causes among PWH. We emphasize the importance of interventions addressing comorbid conditions, cancer management, liver health, and cardiovascular risks. Continuous, systematic data collection and comprehensive monitoring of causes of death remain essential to enable tailored interventions and bridge the ongoing health disparities between PWH and the general population.

## Supplementary Data

Supplementary materials are available at *Clinical Infectious Diseases* online. Consisting of data provided by the authors to benefit the reader, the posted materials are not copyedited and are the sole responsibility of the authors, so questions or comments should be addressed to the corresponding author.

## Supplementary Material

ciae014_Supplementary_Data

## Notes

***Author Contributions***. All coauthors have reviewed and approved the manuscript and made significant contributions to this work.

***Acknowledgments***. The authors thank the participants of the Swiss HIV Cohort Study (SHCS); the physicians, and study nurses for excellent patient care; the SHCS data center and K. Benic for excellent data management; and D. Perraudin and M. Amstad for administrative assistance. The data were gathered by 5 Swiss university hospitals, 2 cantonal hospitals, 16 affiliated hospitals, and 45 private physicians (listed in http://www.shcs.ch/180-health-care-providers).

***SHCS members.*** I. Abela, K. Aebi-Popp, A. Anagnostopoulos, M. Battegay, E. Bernasconi, D. L. Braun, H. C. Bucher, A. Calmy, M. Cavassini, A. Ciuffi, G. Dollenmaier, M. Egger, L. Elzi, J. Fehr, J. Fellay, H. Furrer, C. A. Fux, H. F. Günthard (SHCS president), A. Hachfeld, D. Haerry (deputy of Positive Council), B. Hasse, H. H. Hirsch, M. Hoffmann, I. Hösli, M. Huber, D. Jackson-Perry (patient representative), C. R. Kahlert (chairman of the Mother & Child Substudy), O. Keiser, T. Klimkait, R. D. Kouyos, H. K., K. Kusejko (head of Data Centre), N. Labhardt, K. Leuzinger, B. Martinez de Tejada, C. Marzolini, K. J. Metzner, N. Müller, J. Nemeth, D. Nicca, J. Notter, P. Paioni, G. Pantaleo, M. Perreau, A. Rauch (chairman of the Scientific Board), L. Salazar-Vizcaya, P. Schmid, R. Speck, M. Stöckle (chairman of the Clinical and Laboratory Committee), P. Tarr, A. Trkola, G. Wandeler, M. Weisser, and S. Yerly.

***Manuscript drafting***. The authors initially drafted the manuscript. ChatGPT (GPT-3, last accessed 23 October 2023) from OpenAI was later used to correct grammar correction and enhance language clarity. ChatGPT was not involved in analysis, content creation, or literature review. The authors meticulously reviewed, verified, and edited any content generated by ChatGPT, taking complete responsibility for the manuscript's quality and accuracy.

***Financial support***. This work has been financed within the framework of the SHCS and supported by the Swiss National Science Foundation (grant 201369), by SHCS project 916 and the SHCS Research Foundation, as well as the Swiss Federal Office for Public Health.
