# Supplementary material for: Time Trends in Causes of Death in People With HIV: Insights From the Swiss HIV Cohort Study
Source: Clin Infect Dis. 2024 Jan 12;79(1):177–88. doi: 10.1093/cid/ciae014 (PMC11259222; doi:10.1093/cid/ciae014)
Supplement: ciae014_Supplementary_Data [file ciae014_supplementary_data.docx]

Supplementary Material:

***Time Trends in Causes of Death in People with HIV: Insights from the Swiss HIV Cohort Study***

Authors:

M.S.R. Weber^1,2^; J.J. Duran Ramirez^1,2^; M. Hentzien^3,4,5^; M. Cavassini^6,7^; E. Bernasconi^8,9^; E. Hofmann^10^; H. Furrer^10^; H. Kovari^11^; M. Stöckle^12^; P. Schmid^13^; D. Haerry^14^; D.L. Braun*^1,2^; H.F. Günthard*^1,2^; K. Kusejko*^1,2^; Swiss HIV Cohort Study**

*Authors D.L. Braun, H.F. Günthard and K. Kusejko contributed equally to this manuscript

**Team members of the Swiss HIV Cohort Study are listed in the acknowledgments

Affiliations

^1^Department of Infectious Diseases and Hospital Epidemiology, University Hospital Zurich, Zurich, Switzerland, ^2^ Institute of Medical Virology, University of Zurich, Zurich, Switzerland, ^3^HIV/AIDS unit, Department of Infectious Diseases, University Hospital Geneva, Geneva, Switzerland, ^4^University of Geneva, Geneva, Switzerland, ^5^Reims Champagne-Ardenne University, Reims, France, ^6^Department of Infectious Diseases, Lausanne University Hospital, Lausanne, Switzerland, ^7^University of Lausanne, Lausanne, Switzerland, ^8^Department of Infectious Diseases, Regional Hospital Lugano EOC, Lugano, Switzerland, ^9^University of Geneva and University of Southern Switzerland, Lugano, Switzerland, ^10^Department of Infectious Diseases, Inselspital, Bern University Hospital, University of Bern, Bern, Switzerland, ^11^Center for Infectious Diseases, Klinik im Park, Zürich, Switzerland, ^12^Division of Infectious Diseases and Hospital Epidemiology, University Hospital Basel, University of Basel, Basel, Switzerland, ^13^Division of Infectious Diseases and Hospital Epidemiology, Cantonal Hospital St. Gallen, St. Gallen, Switzerland, ^14^Positive Council Switzerland, Zürich, Switzerland

Short Summary

We analyzed death causes in the Swiss HIV Cohort Study (2005-2022). HIV/AIDS-related and liver-related deaths decreased proportionally, whereas cardiovascular-related deaths remained stable, and non-AIDS cancers increased, underscoring the need for holistic healthcare strategies addressing comorbidities, cancer management, and cardiovascular risks.

Corresponding Author:

Marc Weber

marc.weber4@uzh.ch

Klinik für Infektionskrankheiten und Spitalhygiene,

Universitätsspital Zürich,

Rämistrasse 100,

Zürich, Switzerland,

Supplementary

**Table S1**

| **Table S1: Patient Characteristics by Cause of Death** | | | | | | | | | | | | | | | |
| --- | --- | --- | --- | --- | --- | --- | --- | --- | --- | --- | --- | --- | --- | --- | --- |
| **Patient Characteristics** | **Overall** | **HIV/AIDS** | **Non-AIDS-infection** | **Non-AIDS, non-hepatic Cancer** | **Liver** | **Cardiovascular** | **Respiratory** | **Susbstance Abuse** | **Violent Death** | **CNS** | **Renal/ Urogenital** | **GIT** | **Suicide** | **Unknown/ Unclassifiable** | **Other** |
| **Total (n)** | 1630 | 147 | 101 | 373 | 166 | 158 | 38 | 71 | 53 | 15 | 19 | 22 | 100 | 348 | 19 |
| **Age at time of death, years (median [IQR])** | 54.00 [46.00, 63.00] | 48.00 [40.50, 56.50] | 52.00 [43.00, 68.00] | 58.00 [51.00, 65.00] | 49.50 [44.00, 55.00] | 59.50 [49.00, 71.00] | 60.00 [54.50, 64.00] | 44.00 [40.00, 48.00] | 50.00 [46.00, 61.00] | 75.00 [59.00, 78.50] | 69.00 [64.50, 78.50] | 52.00 [46.50, 58.50] | 50.00 [43.75, 60.00] | 54.00 [47.00, 63.00] | 71.00 [56.50, 73.50] |
| **Follow-up time, years (median [IQR])** | 13.39 [7.53, 19.74] | 8.07 [1.66, 15.18] | 14.29 [8.99, 22.19] | 15.01 [9.01, 21.80] | 15.26 [9.73, 19.87] | 13.26 [8.58, 19.69] | 18.06 [12.16, 24.92] | 12.11 [6.57, 15.05] | 15.50 [9.26, 20.59] | 12.68 [9.06, 22.51] | 11.04 [7.04, 19.45] | 14.72 [7.10, 23.54] | 13.27 [8.15, 20.07] | 12.35 [6.32, 18.09] | 10.27 [7.38, 18.94] |
| **Assigned female at birth (%)** | 386 (23.7) | 39 (26.5) | 32 (31.7) | 99 (26.5) | 35 (21.1) | 33 (20.9) | 8 (21.1) | 21 (29.6) | 7 (13.2) | 3 (20.0) | 5 (26.3) | 5 (22.7) | 12 (12.0) | 82 (23.6) | 5 (26.3) |
| **HIV acquisition mode (%)** |  |  |  |  |  |  |  |  |  |  |  |  |  |  |  |
| **Men who have sex with men** | 506 (31.0) | 50 (34.0) | 21 (20.8) | 120 (32.2) | 19 (11.4) | 70 (44.3) | 15 (39.5) | 15 (21.1) | 13 (24.5) | 8 (53.3) | 6 (31.6) | 7 (31.8) | 57 (57.0) | 98 (28.2) | 7 (36.8) |
| **Heterosexual contact** | 488 (29.9) | 41 (27.9) | 24 (23.8) | 151 (40.5) | 31 (18.7) | 51 (32.3) | 10 (26.3) | 7 (9.9) | 20 (37.7) | 3 (20.0) | 8 (42.1) | 8 (36.4) | 15 (15.0) | 112 (32.2) | 7 (36.8) |
| **People who inject drugs** | 571 (35.0) | 47 (32.0) | 50 (49.5) | 86 (23.1) | 115 (69.3) | 32 (20.3) | 12 (31.6) | 48 (67.6) | 16 (30.2) | 3 (20.0) | 3 (15.8) | 6 (27.3) | 23 (23.0) | 128 (36.8) | 2 (10.5) |
| **Other** | 65 (4.0) | 9 (6.1) | 6 (5.9) | 16 (4.3) | 1 (0.6) | 5 (3.2) | 1 (2.6) | 1 (1.4) | 4 (7.5) | 1 (6.7) | 2 (10.5) | 1 (4.5) | 5 (5.0) | 10 (2.9) | 3 (15.8) |
| **Ethnicity (%)** |  |  |  |  |  |  |  |  |  |  |  |  |  |  |  |
| **White** | 1499 (92.0) | 125 (85.0) | 89 (88.1) | 343 (92.0) | 159 (95.8) | 148 (93.7) | 37 (97.4) | 68 (95.8) | 49 (92.5) | 15 (100.0) | 15 (78.9) | 19 (86.4) | 93 (93.0) | 322 (92.5) | 17 (89.5) |
| **Black** | 74 (4.5) | 10 (6.8) | 10 (9.9) | 15 (4.0) | 3 (1.8) | 6 (3.8) | 1 (2.6) | 1 (1.4) | 2 (3.8) | 0 (0.0) | 3 (15.8) | 2 (9.1) | 4 (4.0) | 16 (4.6) | 1 (5.3) |
| **Hispano-American** | 15 (0.9) | 6 (4.1) | 0 (0.0) | 4 (1.1) | 1 (0.6) | 2 (1.3) | 0 (0.0) | 1 (1.4) | 1 (1.9) | 0 (0.0) | 0 (0.0) | 0 (0.0) | 0 (0.0) | 0 (0.0) | 0 (0.0) |
| **Asian** | 25 (1.5) | 3 (2.0) | 1 (1.0) | 8 (2.1) | 2 (1.2) | 2 (1.3) | 0 (0.0) | 1 (1.4) | 0 (0.0) | 0 (0.0) | 1 (5.3) | 1 (4.5) | 3 (3.0) | 2 (0.6) | 1 (5.3) |
| **Other / Unknown** | 17 (1.0) | 3 (2.0) | 1 (1.0) | 3 (0.8) | 1 (0.6) | 0 (0.0) | 0 (0.0) | 0 (0.0) | 1 (1.9) | 0 (0.0) | 0 (0.0) | 0 (0.0) | 0 (0.0) | 8 (2.3) | 0 (0.0) |
| **Higher Education or University degree (%)** | 314 (19.3) | 31 (21.1) | 17 (16.8) | 82 (22.0) | 15 (9.0) | 39 (24.7) | 4 (10.5) | 5 (7.0) | 10 (18.9) | 3 (20.0) | 6 (31.6) | 6 (27.3) | 30 (30.0) | 61 (17.5) | 5 (26.3) |
| **Years since HIV diagnosis (median [IQR])** | 17.00 [11.00, 24.00] | 12.00 [6.00, 19.50] | 18.00 [12.00, 24.00] | 19.00 [11.00, 25.00] | 19.00 [14.00, 23.00] | 16.00 [10.00, 23.00] | 22.00 [16.50, 25.75] | 14.00 [8.00, 19.50] | 17.00 [12.00, 22.00] | 14.00 [10.00, 24.50] | 19.00 [9.50, 23.50] | 20.00 [12.25, 26.00] | 17.00 [10.00, 22.25] | 17.00 [11.00, 24.00] | 11.00 [8.00, 22.00] |
| **CD4 Nadir (median [IQR])** | 128.00 [48.00, 230.00] | 52.00 [18.00, 151.00] | 120.00 [57.00, 194.00] | 132.00 [52.00, 218.00] | 95.50 [32.00, 193.75] | 144.00 [55.50, 243.50] | 128.00 [55.50, 212.25] | 139.00 [76.00, 248.00] | 125.00 [74.00, 225.00] | 193.00 [104.50, 282.50] | 144.00 [67.50, 237.00] | 114.00 [50.50, 247.25] | 176.00 [93.50, 260.75] | 141.00 [57.25, 260.00] | 195.50 [80.75, 271.75] |
| **Prior clincial AIDS (%)** | 673 (41.3) | 117 (79.6) | 42 (41.6) | 145 (38.9) | 71 (42.8) | 51 (32.3) | 18 (47.4) | 18 (25.4) | 16 (30.2) | 7 (46.7) | 8 (42.1) | 14 (63.6) | 36 (36.0) | 124 (35.6) | 6 (31.6) |
| **Years on ART (median [IQR])** | 13.00 [8.00, 19.00] | 9.00 [3.00, 13.00] | 14.00 [10.00, 19.75] | 14.00 [9.00, 20.00] | 13.00 [9.00, 17.00] | 13.00 [9.00, 19.75] | 16.00 [12.00, 20.00] | 10.00 [7.00, 13.00] | 15.00 [9.00, 18.00] | 13.00 [6.50, 16.00] | 13.00 [9.00, 19.50] | 12.00 [9.00, 16.75] | 12.00 [8.75, 18.00] | 13.00 [8.00, 19.00] | 12.50 [7.25, 20.00] |
| **On ART at time of death (%)** | 985 (60.4) | 78 (53.1) | 63 (62.4) | 269 (72.1) | 101 (60.8) | 107 (67.7) | 28 (73.7) | 45 (63.4) | 28 (52.8) | 12 (80.0) | 14 (73.7) | 18 (81.8) | 51 (51.0) | 160 (46.0) | 11 (57.9) |
| **Smoked, ever (%)** | 1265 (77.6) | 102 (69.4) | 78 (77.2) | 287 (76.9) | 147 (88.6) | 120 (75.9) | 36 (94.7) | 70 (98.6) | 44 (83.0) | 8 (53.3) | 10 (52.6) | 17 (77.3) | 71 (71.0) | 263 (75.6) | 12 (63.2) |
| **Hypertension (%)** | 920 (56.4) | 45 (30.6) | 57 (56.4) | 217 (58.2) | 102 (61.4) | 125 (79.1) | 28 (73.7) | 28 (39.4) | 31 (58.5) | 11 (73.3) | 17 (89.5) | 12 (54.5) | 44 (44.0) | 190 (54.6) | 13 (68.4) |
| **Body mass index, kg/m2 (median [IQR])** | 22.39 [19.49, 25.36] | 21.26 [18.77, 24.21] | 22.67 [19.29, 25.93] | 22.06 [19.22, 25.54] | 21.81 [19.29, 25.62] | 23.28 [20.88, 25.95] | 19.63 [17.29, 25.29] | 21.96 [19.66, 23.29] | 22.58 [19.68, 25.20] | 23.11 [21.24, 24.34] | 19.57 [17.25, 23.00] | 22.29 [19.53, 25.00] | 23.08 [20.76, 26.20] | 22.79 [19.75, 25.56] | 23.55 [21.02, 26.69] |
| **Diabetes mellitus (%)** | 195 (12.0) | 6 (4.1) | 15 (14.9) | 46 (12.3) | 20 (12.0) | 35 (22.2) | 4 (10.5) | 2 (2.8) | 2 (3.8) | 3 (20.0) | 7 (36.8) | 6 (27.3) | 6 (6.0) | 43 (12.4) | 0 (0.0) |
| **Prior cardiovascular event (%)** | 230 (14.1) | 6 (4.1) | 11 (10.9) | 52 (13.9) | 9 (5.4) | 65 (41.1) | 7 (18.4) | 2 (2.8) | 7 (13.2) | 5 (33.3) | 7 (36.8) | 3 (13.6) | 10 (10.0) | 43 (12.4) | 3 (15.8) |
| **Dyslipidemia (%)** | 967 (59.3) | 60 (40.8) | 53 (52.5) | 275 (73.7) | 63 (38.0) | 128 (81.0) | 28 (73.7) | 25 (35.2) | 31 (58.5) | 12 (80.0) | 14 (73.7) | 13 (59.1) | 58 (58.0) | 196 (56.3) | 11 (57.9) |
| **Depression (%)** | 574 (35.2) | 29 (19.7) | 35 (34.7) | 132 (35.4) | 54 (32.5) | 53 (33.5) | 17 (44.7) | 35 (49.3) | 20 (37.7) | 9 (60.0) | 7 (36.8) | 7 (31.8) | 47 (47.0) | 122 (35.1) | 7 (36.8) |
| **HCV co-infection (%)** | 524 (32.1) | 43 (29.3) | 38 (37.6) | 70 (18.8) | 135 (81.3) | 32 (20.3) | 12 (31.6) | 39 (54.9) | 16 (30.2) | 2 (13.3) | 8 (42.1) | 6 (27.3) | 20 (20.0) | 101 (29.0) | 2 (10.5) |
| **HBV co-infection (%)** | 137 (8.4) | 12 (8.2) | 6 (5.9) | 30 (8.0) | 34 (20.5) | 8 (5.1) | 0 (0.0) | 6 (8.5) | 3 (5.7) | 1 (6.7) | 1 (5.3) | 2 (9.1) | 9 (9.0) | 23 (6.6) | 2 (10.5) |
| **CMV co-infection (%)** | 1304 (80.0) | 115 (78.2) | 77 (76.2) | 309 (82.8) | 117 (70.5) | 127 (80.4) | 33 (86.8) | 57 (80.3) | 40 (75.5) | 14 (93.3) | 13 (68.4) | 16 (72.7) | 83 (83.0) | 286 (82.2) | 17 (89.5) |
| Patients' basic, clinical, and laboratory characteristics overall and stratified by broader categories of causes of death, with the definition of all variables found in Table 2 and categorization of single causes into the broader causes found in Table 1, Abbreviations: IQR, interquartile range; HIV, human immunodeficiency virus; AIDS, acquired immunodeficiency syndrome; ART, antiretroviral therapy; BMI, body mass index; HCV, hepatitis C virus; HBV, hepatitis B virus; CMV, cytomegalovirus; NANH, non-AIDS, non-hepatic; CNS, Central nervous system; GIT, Gastrointestinal Tract. | | | | | | | | | | | | | | | |

**Table S2**

| **Table S2: Patient Characteristics of HIV/AIDS-related deaths (2020-2022)** | |
| --- | --- |
| **Patient Characteristics** | **Overall** |
| **Total (n)** | 10 |
| **Age at time of death, years (median [IQR])** | 55.00 [53.25, 60.00] |
| **Follow-up time, years (median [IQR])** | 16.96 [2.04, 24.67] |
| **Assigned female at birth (%)** | 2 (20.0) |
| **HIV acquisition mode (%)** |  |
| **Men who have sex with men** | 6 (60.0) |
| **Heterosexual contact** | 2 (20.0) |
| **People who inject drugs** | 2 (20.0) |
| **Ethnicity = white (%)** | 10 (100.0) |
| **Higher Education or University degree (%)** | 3 (30.0) |
| **Cause of death = Lymphoma (%)** | 8 (80.0) |
| **Years since HIV diagnosis (median [IQR])** | 24.50 [8.75, 30.75] |
| **CD4 Nadir (median [IQR])** | 33.00 [27.25, 109.00] |
| **Prior clincial AIDS (%)** | 8 (80.0) |
| **Years on ART (median [IQR])** | 19.50 [8.50, 24.75] |
| **On ART at time of death (%)** | 8 (80.0) |
| **Smoked, ever (%)** | 5 (50.0) |
| **Hypertension (%)** | 6 (60.0) |
| **Body mass index, kg/m2 (median [IQR])** | 22.14 [18.90, 26.96] |
| **Diabetes mellitus (%)** | 10 (100.0) |
| **Prior cardiovascular event (%)** | 10 (100.0) |
| **Hypercholesterolaemia (%)** | 6 (60.0) |
| **Depression (%)** | 6 (60.0) |
| **HCV co-infection (%)** | 3 (30.0) |
| **HBV co-infection (%)** | 1 (10.0) |
| **CMV co-infection (%)** | 7 (70.0) |
| Patients' basic, clinical, and laboratory characteristics of all ten participants who died of an HIV/AIDS-related underlying cause of death between 2020 and 2022, with the definition of all variables found in Table 2. Abbreviations: IQR, interquartile range; HIV, human immunodeficiency virus; AIDS, acquired immunodeficiency syndrome; ART, antiretroviral therapy; BMI, body mass index; HCV, hepatitis C virus; HBV, hepatitis B virus; CMV, cytomegalovirus. | |

**Figure 1S**

**
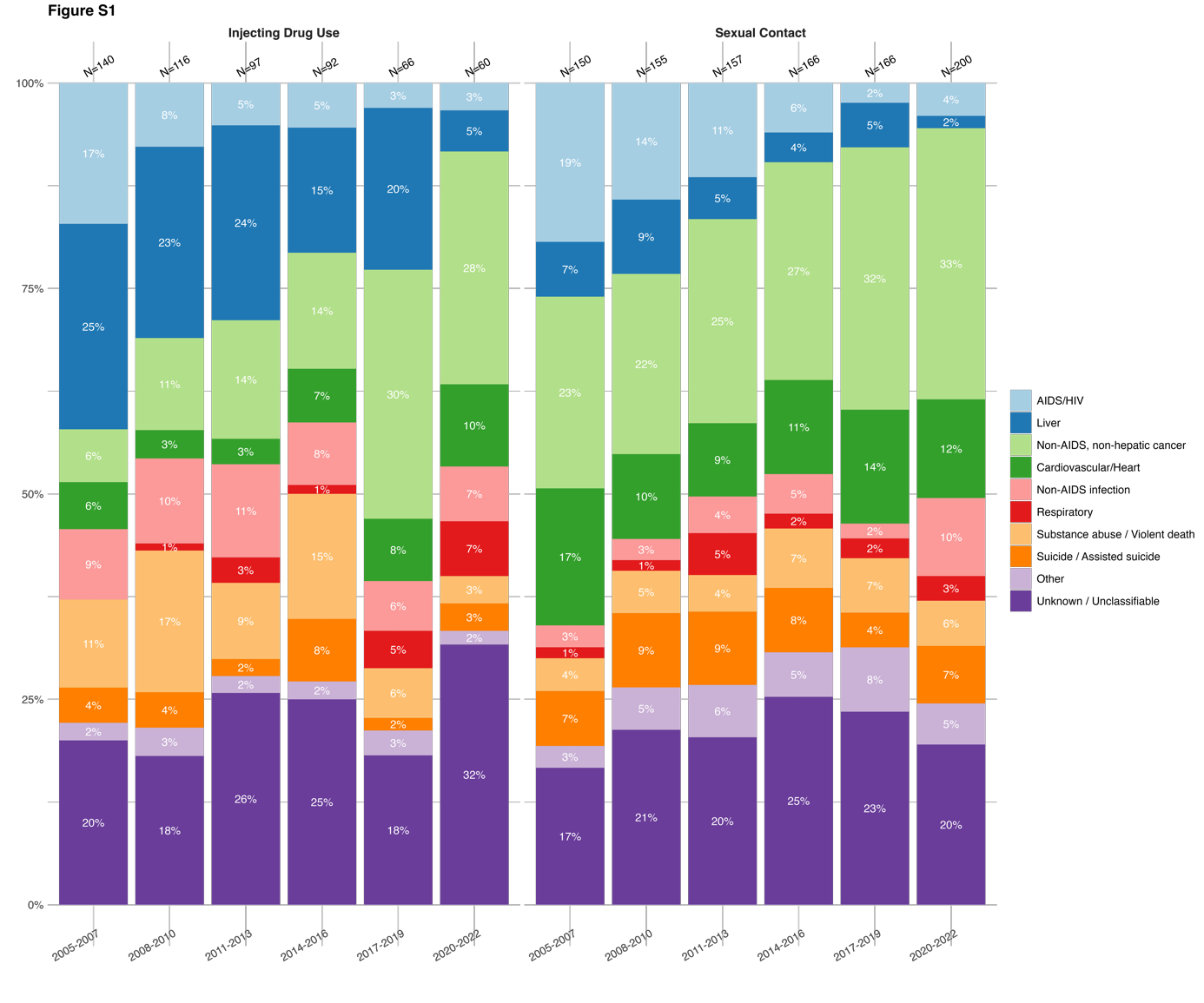
**

**Figure S1: Time trends in causes of death grouped by HIV acquisition mode from 2005 to 2022, stratified by 3-year periods.**  Single causes of death are categorized into broader categories as outlined in Table 1. X-axis; time periods from 2005 to 2022, grouped into 3-year intervals. Y-axis; percentage distribution of each cause of death category. The number above each bar denotes the total reported deaths for the corresponding 3-year period. Bars are color-coded by causes of death as shown in the legend, and the number within each bar represents the percentage of each cause of death category within its respective 3-year period. Abbreviations: HIV, Human Immunodeficiency Virus; AIDS, Acquired Immunodeficiency Syndrome.

**
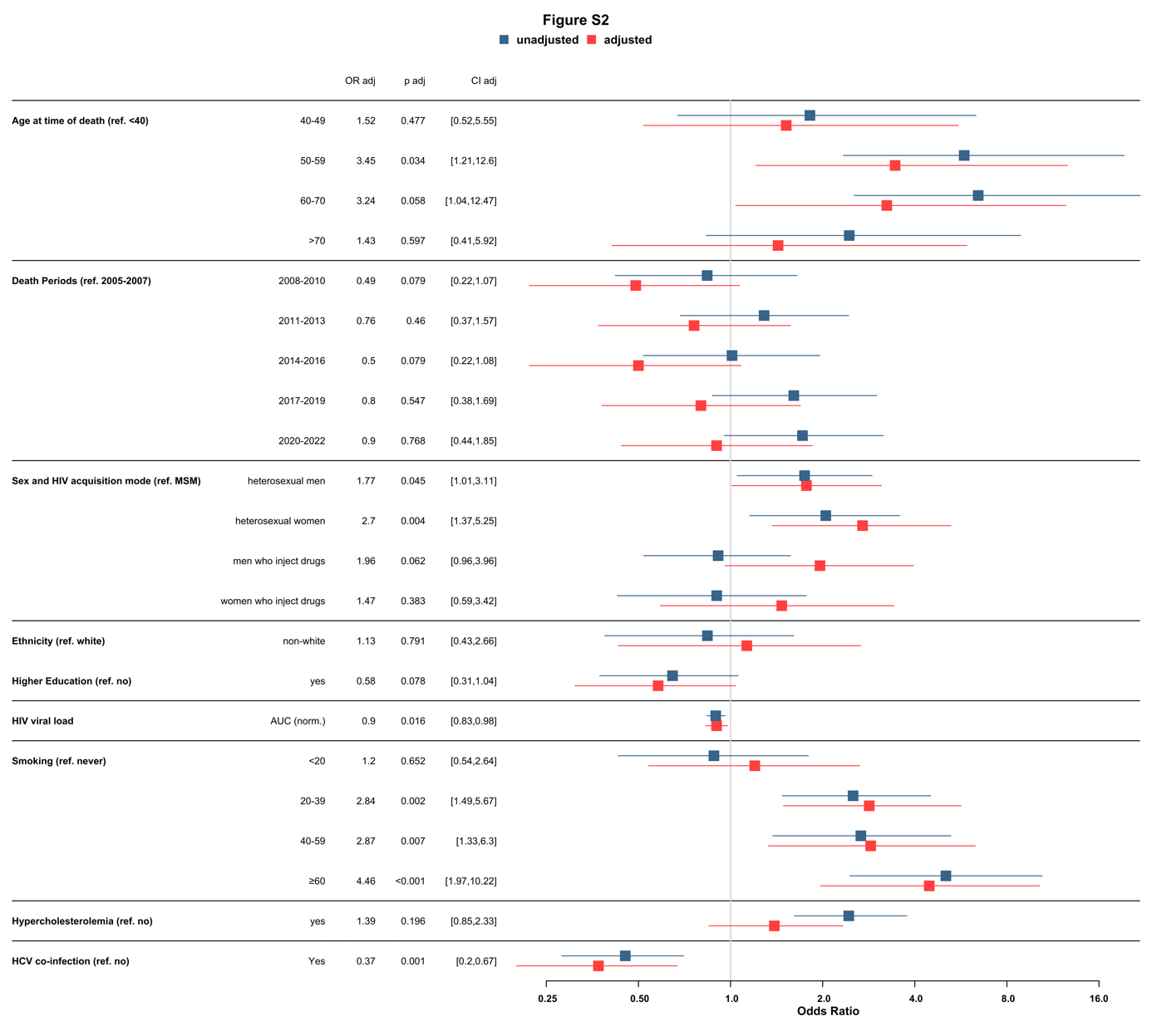
**

**Figure S2:** **Factors associated with lung cancer-related causes of death.** Y-axis; all factors included in the multivariable/adjusted logistic regression analysis (in red) based on their statistical significance in the univariable/unadjusted logistic regression analysis (in blue) and clinical relevance. X-axis; odds ratio of each factor compared to its reference factor (in brackets) of dying from a lung cancer-related cause of death compared to any other causes of death. Abbreviations: HIV, Human Immunodeficiency Virus; AIDS, Acquired Immunodeficiency Syndrome; ART, Antiretroviral Therapy; MSM, Men who have sex with men; IDU, Injecting Drug Use; OR, Odds Ratio; AUC, Area under the curve.
